# Barriers and facilitators to diabetes prevention support for women in Malaysia with gestational diabetes mellitus: A qualitative study

**DOI:** 10.1016/j.pecinn.2025.100438

**Published:** 2025-10-06

**Authors:** Irmi Zarina Ismail, Madeleine Benton, Hafizah Mahamad Sobri, Anisah Baharom, Nicola Guess, Kimberley Goldsmith, Iklil Iman, Siew Mooi Ching, Barakatun-Nisak Mohd Yusof, Nurul Iftida Basri, Mazatulfazura Sf Binti Salim, Faezah Hassan, Helen Murphy, Angus Forbes, Khalida Ismail, Boon How Chew, Iliatha Papachristou Nadal

**Affiliations:** a Department of Psychological Medicine, King's College London, London, UK; b Department of Family Medicine, Faculty of Medicine and Health Sciences, Universiti Putra Malaysia, Selangor, Malaysia.; c Department of Biostatistics & Health Informatics, King's College London, London, UK; d Division of Care for Long Term Conditions, King's College London, London, UK; e Department of Medicine, University of East Anglia, Norfolk, UK; f Research Centre for Optimal Health, University of Westminster, London, UK; g Department of Nutrition and Dietetics, Faculty of Medicine and Health Sciences, Universiti Putra Malaysia, Selangor, Malaysia; h Department of Community Health, Faculty of Medicine and Health Sciences, Universiti Putra Malaysia, 43400 Serdang, Selangor, Malaysia; i Department of Obstetrics and Gynaecology, Faculty of Medicine and Health Sciences, Universiti Putra Malaysia, 43400 Serdang, Selangor, Malaysia; j Department of Rehabilitation Medicine, Faculty of Medicine and Health Sciences, Universiti Putra Malaysia, 43400 Serdang, Malaysia; k Clinical Research Unit, Hospital Pengajar Universiti Putra Malaysia (HPUPM, Teaching Hospital), Persiaran Mardi-UPM, 43400 Serdang, Malaysia

**Keywords:** Prevention of diabetes, Gestational diabetes, Malaysia, Qualitative methods, Womens health

## Abstract

**Objectives:**

To explore the barriers and facilitators to diabetes prevention support among pregnant women in Malaysia.

**Design:**

A qualitative study using individual in-depth interviews, analysed using reflexive thematic analysis.

**Setting:**

Public health clinics and one central government hospital in Selangor, Malaysia.

**Participants:**

Women diagnosed with gestational diabetes mellitus.

**Results:**

Sixteen women aged 26 to 41 years, from three different ethnic groups (Malay, Chinese, and Indian) participated in the study. Key barriers to diabetes prevention support included limited access to relevant and usable information; competing health needs during pregnancy; social and cultural priorities overriding self-care; and structural constraints. Facilitators included intrinsic motivation and self-awareness; awareness of future diabetes risk; social and family support; access to trusted and culturally relevant information. All participants reported challenges in maintaining healthy lifestyle practices, particularly related to physical activity and diet during pregnancy. Information on diabetes prevention support was obtained from various sources, including healthcare providers, online platforms, and family members.

**Conclusion:**

The findings from this study have significant implications for enhancing diabetes prevention support and informing the potential development of tailored diabetes prevention interventions for women in Malaysia.

## Highlights

• Gestational diabetes mellitus is a common pregnancy condition, especially among South Asian women in Malaysia
• We found barriers and facilitators influencing diabetes prevention support for diverse pregnant women groups.
• In Selangor, women with GDM lack adequate information from health providers about effective diabetes prevention.
• Online culturally tailored programs can increase engagement in physical activity and proper dietary management.
• Social and family environments are critical in shaping behavioural changes among women during pregnancy.

## 1 Introduction

Gestational diabetes mellitus (GDM) is one of the most common conditions in pregnancy. The global prevalence of GDM varies considerably by country from 2 % to 38 % [1] and is higher in certain ethnic groups, particularly among South Asian women. Malaysia is one of the countries in Southeast Asia with high GDM burden; with prevalence estimates ranging from 10.3 % to 27.9 % [[2], [3], [4]]. Women with GDM have a significantly higher risk of caesarean section, hypertension, preeclampsia, and shoulder dystocia, compared with the non-GDM population [5]. Moreover, it is associated with a tenfold increased risk of developing type 2 diabetes (T2D) within 10 years after birth [6], and up to 70 % of women will develop impaired glucose regulation [7]. Women with GDM are also more likely to have GDM recurrence in future pregnancies [8]. Therefore, there is considerable need to develop effective interventions to reduce the risk of future GDM and type 2 diabetes mellitus.

Lifestyle modification including to physical activity and diet can prevent or delay future diabetes. A sub-analysis of the large national US Diabetes Prevention Programme demonstrates a 53 % risk reduction of incident type 2 diabetes mellitus in women with a history of GDM [9]. A recent systematic review of lifestyle-based diabetes prevention interventions (DPI) for women with a history of GDM, reported an overall 25 % risk reduction for incident type 2 diabetes mellitus.

The estimated risk reduction for DPIs conducted within six months postpartum was greater at 39 % (RR = 0.61 vs. 1.00; *p* = 0.11) [10]. While current evidence for DPIs in this population is still equivocal, delivering diabetes management and diabetes prevention support (DPS) starting in pregnancy, when women are more engaged with healthcare providers, and continuing into the early postpartum period may improve effectiveness.

A review of existing guidelines concluded that for women with GDM, the most effective physical activity support as first-line treatment, alongside dietary modifications, includes aerobic exercise such as walking, jogging, running, cycling, swimming, elliptical training, or aqua aerobics. This should be performed 3–4 times per week, totalling 50–150 min per week, with a maximum of 30 min per session, in addition to resistance training at least twice weekly [11]. Including such evidence-based DPS is therefore an important component of a DPI. A first step in establishing DPS and the subsequent DPI is to understand how receptive women with GDM are to this support by exploring their experiences.

Currently, little is known about the experiences of DPS in Malaysian women with GDM especially during the antenatal period. The limited data on the effectiveness of lifestyle based DPS for women with GDM in Malaysia is conflicting [12,13]. The inconsistent impact of these studies may reflect some the factors that can mediate women's engagement with advice and support to lifestyle changes in this period. There is a growing body of knowledge demonstrated that women face challenges in addressing their own personal health needs during and after a GDM pregnancy, including: the focus on the health of their baby; emotional responses to GDM (guilt and shame); carer-burden; family/partner support; and problematic interactions with health professionals [14]. This qualitative study is part of a larger research program aimed at preventing future diabetes risk. Because women's experiences and the factors that influence their behaviour are socio-culturally specific in Malaysia, it is important to gain an in-depth understanding of their perspectives [15]. This study explored the lived experiences of Malaysian women with GDM in accessing and using lifestyle-based diabetes prevention support (DPS) during pregnancy. Specifically, it examined perceived facilitators and barriers to the utility and accessibility of DPS, with the aim of informing the development of a culturally and contextually appropriate diabetes prevention intervention (DPI) for this population.

## 2 Materials and methods

This study employed an exploratory descriptive qualitative design to explore the experiences of Malaysian women with GDM in accessing lifestyle-based DPS. As this qualitative study forms part of a larger programme specifically aimed at developing a culturally tailored DPI for Malaysian women with GDM during the antenatal and postnatal periods [15], an exploratory descriptive qualitative design was chosen. This enables us to generate in-depth, contextual insights that can inform the development of culturally and contextually appropriate interventions.

### 2.1 Study setting

Malaysia was selected as the study site because it has one of the highest reported prevalence rates of GDM in Southeast Asia (∼30 %), with higher rates observed in urban populations [16,17]**.** The study was conducted in three urban public health clinics in Selangor, Malaysia's most populous state, to capture experiences in high-prevalence urban settings.

Ethical approval was obtained from the Malaysian Medical Research and Ethics Committee (NMRR-20-750-53,235). Reporting was guided by the Consolidated Criteria for Reporting Qualitative Research (COREQ) [18] (S1 File).

### 2.2 Participants and recruitment

Women were recruited using purposive sampling between September 2020 and April 2021 from three urban public health clinics, all located in Selangor, Malaysia's most populous state with a population of nearly seven million people. Women who were currently pregnant and diagnosed with GDM were purposively sampled from the diabetes registry. Purposive sampling was employed to ensure the inclusion of participants who could provide rich, insightful data regarding their experiences with DPS. Eligible participants were identified through the registry and invited to participate by the research team. This approach allowed us to capture in-depth perspectives from those most directly experiencing the phenomenon under study. There are three main ethnic groups in Selangor, of which 61 % are Malay, 27 % Chinese and 11 % Indian [19]. Potential eligible women were identified from GDM registries and during clinic visits. Women were approached and then screened based on eligibility criteria. Eligible women were invited to an interview at the same facility where they receive medical care. During the movement control order (MCO) in Malaysia due to the COVID-19 pandemic, identified participants were contacted via phone to further confirm eligibility and availability for either on-site or online interview.

The eligibility criteria were women aged ≥18 years; pregnant with GDM; and did not have any serious physical or mental illness. The diagnosis of GDM was in accordance with the 2017 Clinical Practice Guideline: Diabetes in Pregnancy by Ministry of Health Malaysia [20]. Women were purposively sampled for maximum variation, focusing on ethnicity and parity. Written informed consent (S2 File: Consent form) was gained and sociodemographic data was collected prior to the interview. Interviews were scheduled according to the women's availability. We continued to recruit women until data saturation was achieved.

### 2.3 Data collection

Interviews were conducted between September 2020 and April 2021. Six interviews were conducted in person at the clinics and 10 interviews were conducted online using Microsoft (MS) Teams. A following four women gave consent to be interviewed in person but did not attend without giving a reason. The participant and researcher were the only people present during the interview. Interviews were conducted independently by two female researchers with a clinical background, dietician and family physician with an interest in diabetes prevention (IZI and HAS). Both researchers had several years' experience of conducting qualitative research and were able to build rapport quickly with the participants prior to the interview through discussing the reasons why they are conducting the study and talking through the information sheet and consent form.

IZI and HAS followed an interview topic guide (Table 1) with open-ended questions aimed to elicit women's experiences of any advice, support, or information they had received in respect of diabetes risk or lifestyle advice they received during their current or past pregnancies. The topic guide aimed to prompt participants to consider factors that may inhibit or enable their engagement with risk reducing behaviours and activities. The topic guide was piloted online by IPN with a woman with experience of having GDM, based in the UK and from a Southeast Asian background. There were no changes recommended from the pilot interview and a final review of the topic guide was completed by members of the research team experienced in qualitative research (IMI, HAZ, MD, IPN). All interviews were conducted in Bahasa Malaysia, as requested by the participants. Bahasa Malaysia is the lingua franca of Malaysia, however, in almost all interviews, participants occasionally used certain English words interchangeably within their Malay responses.

**Table 1:** The interview topic guide.

Objectives	Questions
Experiences	Can you share with us your **EXPERIENCE** to reduce the risk of getting diabetes since you are diagnosed with GDM? Or after recent delivery?
Need	What are the **SUPPORT** that you receive to help manage your risk of DM during pregnancy with GDM or after delivery?
Importance	Were the information/ support/ activities **IMPORTANT** for your care to prevent diabetes? Why?
Acceptability	Have you used the information that you receive to manage the care during and after GDM?
Challenges/ barriers	What are your **DIFFICULTIES** when you go through the care?
Facilitators	What has **HELPED** you to go through the care? Have you tried finding **OTHER SOURCES** of information? Are there any **SUGGESTIONS** that would help you to reduce your risk of getting diabetes in the future?

### 2.4 Data analysis

Data collection and analysis were conducted concurrently. Inductive reflexive thematic analysis was undertaken [21]. Interview audio recordings were translated from Malay to English by IZI, who is proficient in both languages. Field notes were made by IZI and HAS after each interview.

The transcriptions were reviewed by three authors (IZI, HAS, AN) to ensure quality, confidentiality, reliability, and formatting for coding. All transcripts were managed using qualitative data analysis software (NVivo 12.0). Initial coding was conducted by the first author (IZI), and codes and themes were developed iteratively with the research team, including three Malaysia-based members (IZI, HAS, AN) and two UK-based researchers (MB and IPN) over a series of meetings. The involvement of the UK researchers provided an external perspective to enhance confirmability and reduce potential bias.

To address subjectivity and social desirability bias, the interviewers (IZI and HAS), who are healthcare providers, engaged in reflexivity by reflecting on how their professional backgrounds might shape the interviews and analysis. Participants were reassured about confidentiality, and neutral, open-ended questions were used to minimize influence. Consultation with the clinic dietician helped ensure ethical and unbiased conduct of the interviews.

Disagreements or discrepancies on codes were resolved by re-examining the meaning of the codes in relation to the study objectives. A description of each main and subtheme was used to define the themes, with illustrative quotes serving as evidence. A final report, including a selection of the most compelling extracts and their analysis, was produced by IPN with additional input from authors AF and MD.

## 3 Results

A total of 16 women with current GDM were interviewed via 5 focus group discussions. The age range of participants was between 26 and 41 years, and the majority of women were Malay (*n* = 11), followed by Indian (*n* = 3) and Chinese (*n* = 2) (see Table 2).

**Table 2:** Participant characteristics.

	Participant	Ethnicity	Age	Parity	History of GDM	Reported DPS use
1.	WD01	Malay	38	4th pregnancy	No	Yes
2.	WD02	Malay	36	2nd pregnancy	Yes	Yes
3.	WD03	Malay	40	4th pregnancy	Yes	Yes^MA^
4.	WD04	Chinese	35	3rd pregnancy	Yes	Yes^MA^
5.	WD05	Chinese	33	3rd pregnancy	No	No
6.	WD06	Indian	30	2nd pregnancy 1 miscarriage	No	Yes
7.	WD07	Indian	41	3rd pregnancy	No	No
8.	WD08	Malay	36	3rd pregnancy	No	Yes
9.	WD09	Malay	32	2nd pregnancy	Yes	Yes^MA^
10.	WD10	Malay	38	1st pregnancy	No	No
11.	WD11	Malay	38	6th pregnancy 2 miscarriages	No	Yes^MA^
12.	WD12	Malay	32	2nd pregnancy	No	Yes
13.	WD13	Malay	29	1st pregnancy	No	Yes
14.	WD14	Malay	26	1st pregnancy	No	Yes
15.	WD15	Malay	27	3rd pregnancy 2 miscarriages	No	Yes
16.	WD16	Indian	31	2nd pregnancy	No	Yes

*GDM = Gestational Diabetes Mellitus *DPS = Diabetes Prevention Interventions.

MA - Uses mobile application as diabetic prevention support.

The findings are presented in two sections: (1) barriers experienced in engaging with DPS and (2) the facilitators that influence uptake of DPS (Table 3). From the results, four main themes emerged for the barriers experienced: limited access to relevant and usable information; competing health needs during pregnancy; social and cultural priorities overriding self-care; structural constraints. Four main themes emerged for the facilitators experienced: intrinsic motivation and self-awareness; awareness of future diabetes risk; social and family support; access to trusted and culturally relevant information.

**Table 3:** Barriers and facilitators to engaging with diabetes prevention support (DPS) among women with GDM.

Barriers	Facilitators
Limited access to relevant and usable information	Intrinsic motivation and self-awareness
Competing health needs during pregnancy	Awareness of future diabetes risk
Social and cultural priorities overriding self-care	Social and family support
Structural constraints	Access to trusted and culturally relevant information

### 3.1 Barriers experienced in engaging with DPS

Women discussed several challenges in managing their GDM and in reducing their risk of future diabetes antenatally.

#### 3.1.1 Limited access to relevant and usable information

Many women reported that DPS was not a priority, as work and family responsibilities took precedence. Information materials were available, but women often lacked time or energy to access them. Women described their focus being primarily on managing GDM, with limited understanding of diabetes prevention during this period. Consequently, the potential long-term benefits of learning about diabetes prevention while managing GDM were often overlooked.

“*I don't know. Maybe due to work, I don't have time to look at the phone. I will only look at the phone at night before sleeping. Even then only for a short while. I'm busy with work the whole day because I'm working with the laptop, so I don't have time to browse*.”

(WD15)

Those who attempted to access information online described challenges such as poor internet connectivity, limited phone storage, or difficulty navigating apps.

“*One thing is the internet connection. If there is no internet, and if it wants to run, it will take up the memory*.”

(WD07)

“*I don't really know about this thing (app). I don't know why. Maybe I don't really feel like using it. Maybe I won't download it. Normally I just browse here, in Facebook, that is all.*”

(WD08)

Participants felt that the content of the information available did not clearly communicate their risk of diabetes or the lifestyle changes required to reduce it. They described advice from health professionals as ambiguous, particularly in relation to food choices and portion sizes.

*“Sometimes we may have overlooked the food that we can and cannot eat. And we don't know how much we can take.”*

(WD13)

Women also highlighted that online resources and apps often lacked specific guidance for GDM. While such tools provided lists of foods, they rarely included culturally relevant advice or portion sizes needed for effective diabetes management.

*“Like this app; the Asian parent. It doesn't have for GDM, like how do we make our diet plans… It tells me all the types of food that we can eat but not the amount of the food that we can eat. Like, how many banana fritters we can eat?”*

(WD13)

#### 3.1.2 Competing health needs during pregnancy

Another area of concern for women was the belief that following the recommended diabetes diet would have a negative impact on their health and unborn baby. Women were concerned that dietary restrictions to manage GDM might compromise fetal nutrition or their own health.

*“I don't know how to calculate calorie during pregnancy. I want to reduce weight but at the same time I'm worried whether my child gets enough nutrient or not. It will look like I'm selfish.”*

(WD02)

Some participants also reported challenges managing co-existing conditions such as anaemia, which required them to increase food intake in ways that conflicted with GDM dietary advice.

*“I felt that it is going to be hard if my haemoglobin is low, at the same time I have GDM… I can't eat too much because the weight cannot exceed because of the GDM.”*

(WD08)

Furthermore, tiredness during pregnancy was commonly discussed among women. Women explained a lack of energy to do any other physical activities other than what they thought was necessary and therefore lacked motivation to exercise.

“*There is no real obstacle; I just don't have the strength. I am tired just by walking. Exercise?.., don't think so. The nurses asked me to do some breathing exercises; there are some exercises recommended in the pink book but I didn't do them*.”

(WD02)

While frequent hunger is common in pregnancy, participants described sudden, unpredictable bouts that complicated adherence to recommended diabetes meal plans. This finding is unique because it highlights how typical pregnancy-related hunger can pose a specific challenge for diabetes management, requiring dietary adjustments that may conflict with medical recommendations. Despite some participants having timely meals, others needed to eat more frequently to accommodate these bouts, making it difficult to follow the recommended diabetes diet.

*“I eat every 2–3 hours. Well, if not we will be hungry when we sleep later. I am indeed hungry.”*

(WD09)

#### 3.1.3 Pregnancy-related fatigue and physical limitations

Tiredness during pregnancy was commonly discussed among women. They described a lack of energy and motivation to exercise, despite understanding its benefits.

*“There is no real obstacle; I just don't have the strength. I am tired just by walking. Exercise? don't think so. The nurses asked me to do some breathing exercises; there are some exercises recommended in the pink book but I didn't do them.”*

(WD02)

#### 3.1.4 Social and cultural priorities overriding self-care

Women described difficulty implementing dietary recommendations when they conflicted with family eating habits. Preparing separate meals for themselves was impractical and often led to frustration.

*“If I am the only one who eats what I cooked, it would be boring. I always cook what the children eat. The specialist clinic told me to cook this… to use planta and not oil to stir fry if I am making fried rice. I did it. But maybe too much planta and it's a bit creamy. So, they (kids and husband) didn't eat it.”*

(WD15)

Childcare responsibilities also took precedence over women's own health needs. Participants explained that supporting children's schooling and daily routines left little time or flexibility for exercise and self-care.

*“If there is not enough staff at school, I can't go out as have to be home with the kids. Only when there is enough staff, can I take time to go for my afternoon exercise. I can't go nighttime because my children are all back from school. Need to help them with their homework. So I can only exercise in the afternoon.”*

(WD04)

#### 3.1.5 Structural constraints

Some participants were diagnosed with GDM late in pregnancy, leaving little time to implement lifestyle changes before delivery.

*“… difficult to control it for a month. I was told that I had diabetes week 33 or week 34. Then I already need to go for C-section next month.”*

(WD12)

Others reported difficulties accessing healthy foods, particularly during the COVID-19 movement restrictions, when fresh ingredients were harder to obtain. Even when women knew what they should eat, practical barriers limited their ability to follow recommendations.

*“When we take ‘quarter quarter half’ meal, we need a complete meal, like vegetables and enough protein. Sometimes at home we have enough protein but not vegetables. Can be an issue just to buy them.”*

(WD13)

*“I know what to eat but I don't apply it. Maybe with MCO (movement control order during COVID pandemic) and all, it is not easy to go to market, everything also needs to line up.”*

(WD10)

### 3.2 Facilitators to engaging with DPS

#### 3.2.1 Intrinsic motivation and self-awareness

The women who had a good understanding of their GDM and risk of having future diabetes felt a strong motivation to improve their lifestyle, to ensure good pregnancy outcomes as well as good life.

*“Just think about it, if we don't take care of ourselves, who will take care of us? If we don't change, then what, right? So, it is self-awareness, sort of, right?”*

(WD15)

Body image and prior experiences with weight were important motivators for dietary change. Some women described feeling stigmatised for being overweight, which encouraged them to persist with healthier eating. Others emphasised the value of tailoring physical activity and diet to what felt personally comfortable and culturally familiar. For instance, participants highlighted that exercise videos in their native language, particularly those with music, were motivating and enjoyable. Similarly, women preferred home-cooked meals, which allowed them to estimate portion sizes more easily and ensure the use of fresh, good-quality ingredients. Simplicity and cultural familiarity were seen as essential to sustaining these changes.

*“I follow YouTube exercises in Malay. The music makes me excited, it feels fun and not like I am forced.”*

(WD10)

*“When I cook at home, I can control the portion and choose fresh ingredients. It feels easier that way.”*

(WD07)

#### 3.2.2 Awareness of future diabetes risk

Many women recognised their heightened risk of developing diabetes after pregnancy, which motivated them to adopt healthier behaviours such as following dietary advice and engaging in physical activity. Fear of diabetes becoming a permanent condition, or of needing long-term insulin, was a powerful driver of change. For some, the desire to avoid further complications such as obesity also reinforced adherence to low-sugar or weight-reduction diets.

*“I don't want to be on insulin forever. That thought makes me follow the diet strictly.”*

(WD06)

#### 3.2.3 Social and family support

Spouses, children, and extended family played an important role in encouraging and sustaining lifestyle changes. Women described families reducing the purchase of sweets, eating more vegetables, and exercising together.

*“I can control the food…. but need the support of family, children. When I was first diagnosed with GDM, I informed them, we can't eat out often. Then we can't buy things like chocolate. Need to reduce the quantity. More vegetable and fruits. My children understood why I buy all these… My husband also eats the rice (brown rice).. he will follow what I eat.”*

(WD01)

*“We (husband and I) would go to the park. He jogged. I brisked walked. I can't jog anymore. So I just walk… also when I go brisk walking, I can do two things at once. I can exercise for myself and go for a walk with my son.”*

(WD09)

Supportive work environments, where employers encouraged physical activity, were also mentioned. For example, one participant described that they had a friendly exercise work environment that allowed and encouraged her to be active physically even during pregnancy.

#### 3.2.4 Access to trusted and culturally relevant information

Participants expressed a clear preference for information delivered in their native language and from Asian-based sources, which they perceived as better aligned with their cultural practices, diets, and everyday realities. For many, diabetes prevention measures were only considered relevant if framed within their cultural context. At the same time, women were cautious about online sources, highlighting the need to distinguish between trustworthy and unreliable information.

*“If I feel that the information received is a big contrast and contradicts a few articles, then I will search up more. But generally, the information is more or less the same so I just conclude. If it is a journal article, we could see how many people has cited the page… But we can't know whether a website is genuine and authentic or not.”*

(WD11)

Visual and engaging formats, such as infographics or videos with music, were preferred over text-heavy materials, as they were seen as easier to understand and more motivating.

*“These pages (Instagram) are more infographics. I feel more comfortable reading information through these pages because it has pictures. There are videos sometimes.”*

(WD09)

Women reported greater trust in official websites when seeking information about diabetes. They also expressed a preference for infographics and videos over written materials, as these visual formats were more engaging, easier to understand, and helped sustain their interest.

“*These pages (Instagram) are more infographics. I feel more comfortable reading information through these pages because it has pictures. There are videos sometimes*.”

(WD09)

## 4 Discussion and conclusion

### 4.1 Discussion

This study explored the barriers and facilitators of DPS during pregnancy among 16 women from three different ethnic groups in Malaysia. Through this investigation, four key themes emerged for the barriers experienced: limited access to relevant and usable information; competing health needs during pregnancy, social and cultural priorities overriding self-care, structural constraints.

and four main themes emerged for facilitators experienced: intrinsic motivation and self-awareness, awareness of future diabetes risk; social and family support; access to trusted and culturally relevant information.

To the best of our knowledge, this is the first study to examine the perspectives of women with GDM on DPS in Malaysia. The findings provide valuable insights into the need for enhanced management and support for mothers at risk of diabetes in Malaysia. Findings from this study demonstrates that many women with GDM are unaware of their heightened risk of developing T2D in the future, which reduces their motivation to seek diabetes prevention support or to modify their behaviour both during and after pregnancy. These results align with evidence from two systematic reviews, which found that women with GDM often lack understanding of diabetes risk factors [21] and that very few recognise their high risk of developing diabetes later in life [22].

### 4.2 Malaysia women with GDM and their HCPs

In Malaysia, dietitians play a key role in advising on GDM management and later diabetes prevention [23]. However, during the Covid-19 pandemic visits to dieticians declined, potentially affecting women's understanding of diabetes prevention. A meta-analysis of the effectiveness of diabetes prevention interventions found that those delivered by dietitians resulted in greater weight loss and lower costs compared to those delivered by non-dietitians [24]. Despite this, women in this study felt that the guidance provided by dietitians on lifestyle changes was too general and lacked specificity. This lack of clear guidance led them to seek advice from family members or colleagues who had experience with GDM and lifestyle changes [25].

Healthcare providers may not prioritise DPS during pregnancy, and women have reported a lack of postpartum care regarding diabetes prevention [21]. In some South Asian countries, healthcare professionals acknowledged the challenges of counselling women with GDM and expressed the need for refresher courses to improve the delivery of lifestyle interventions [26]. Therefore, it is crucial for healthcare providers to personalise their advice based on each mother's understanding and capacity to implement changes. Early face-to-face counselling has been suggested as an effective support strategy to help prevent diabetes [21].

### 4.3 Lifestyle behavioural changes

The two main lifestyle initiatives for diabetes prevention support experienced by women in this study were low intensive physical activity (walking/ house chores) and diet control. This supports another study conducted in Malaysia that focus on physical activity during pregnancy and results indicated that women with GDM did only light exercises, which did not meet the requirement of international physical activity questionnaire IPAQ [27]. Although women with GDM believe that exercise can control glucose levels during pregnancy, they take part in less physical activities during pregnancy compared to after pregnancy [28]. The women in this study discussed dietary interventions optimising maternal glycemia and diabetes prevention, which is similar to studies that focused on western countries i.e. United States (US) and Finland [29,30]. As in the US study, women in this study have also reported to compose a meal plan with balanced, low calorie and low glycaemic index diet during pregnancy to reduce risk of diabetes mellitus [29].

### 4.4 Information from trustworthy sources

The study findings showed that women with GDM access a limited range of information regarding DPS, especially when face-to-face support was not provided. Online platforms were perceived to be useful to support self-care and lifestyle with GDM [25]. A systematic review supports this and has shown that diabetes education on lifestyle activities containing digital features were demonstrated most effective to reduce weight [31]. However, one of the main barriers found in this study was with web browsing and not knowing where to access appropriate trustworthy information. Furthermore, the Malaysian women in this study, experienced technical difficulties accessing the internet which is often a deterring encounter faced by e-health users This is also an issue that has been supported by studies in high income counties [25].

### 4.5 Social norms and support

Different cultural belief may dictate DPS differently. One of the common reasons that influence the compliance to DPS is the perceived control beliefs of certain culture especially among Asians [29]. For example, it is believed among the Chinese population that mothers need rest and may require to stop working during this time, hence exercise is not encouraged [28] and that exercise during pregnancy may lead to miscarriage [33]. However, regardless of culture, in this study exercising was carried out only when women felt that they could manage exercising, however commitment to family (e.g. taking care of their children or house chores) usually took priority [14,26].

Women with GDM especially those from Asia have described difficulties adjusting to the recommended diet which is perceived to be less palatable compared to their traditional food [34]. Likewise, women in the current study iterated their difficulty in resisting their usual food. Having to resist traditional food is a recognised barrier for women with GDM [22]. Women with GDM felt that they would lose control of their daily activities and value in life if told to eat food that they are not accustomed to eating [15,32] Providing women with known cultural foods may influence women's perception of a good diet and therefore more likely to change their behaviour towards food. Furthermore, this study shows that women have a limited understanding of DPS when instructions are not displayed in their first language and supporting research indicates this is an important barrier to optimising maternal glycaemia [35]. This current study showed that participants preferred to have information in their native language even though they understood English. This is because they perceived this as being more personalised and therefore more likely to put the information into practice.

### 4.6 Innovation

This research investigates a relatively overlooked area of healthcare in Malaysia by looking into the experiences of women with GDM regarding diabetes prevention. Although there are global studies on GDM, there is a lack of specific literature that addresses the cultural and social factors affecting Malaysian women's experiences. By focusing on these distinct viewpoints, the study enhances the understanding of GDM by considering cultural sensitivities and practices. The use of qualitative interviews as a primary method represents an innovative approach to gather in-depth, personal insights into the challenges faced by women with GDM in Malaysia. This approach has enabled us to hear the participants' stories, providing a deeper understanding of their needs, coping methods, and support networks.

The findings can inform local health policies and practices related to maternal care by emphasising the importance of culturally appropriate interventions. This method highlights the potential for innovation in healthcare delivery, specifically by promoting educational programmes and support networks that align with the cultural values and needs of women in Malaysia. The impact of this research goes beyond the local area, demonstrating how insights from interviews with Malaysian women can be adapted into strategies for similar cultural environments globally. Therefore, the study encourages the use of localised findings to influence wider health practices, making marking an important contribution to the field.

### 4.7 Limitation and strength

This study employed several strategies to enhance the trustworthiness and rigor of the findings. Reflexivity was practiced by the interviewers, who are healthcare providers, to minimize the influence of their professional background on data collection and interpretation. Social desirability bias was mitigated through neutral, open-ended questioning and reassurance of confidentiality. The iterative development of codes and themes involved both Malaysia- and UK-based researchers, providing an external perspective that enhanced confirmability.

Although qualitative findings are not statistically generalisable, they provide valuable insights into the experiences of women with GDM in urban Malaysia. These insights can help healthcare providers understand the need for enhanced diabetes prevention support and management, while also informing the development of culturally appropriate interventions.

### 4.8 Conclusion

This study highlights important factors preventing women with GDM participation in DPS and gives new insights into how women in Malaysia perceived managing risk of diabetes and the support provided both within health care and the individual's social network. Even though women with GDM in Malaysia practised some form of lifestyle initiatives to prevent diabetes, the overall lack of experience and knowledge in diabetes prevention is very low. Women showed effort in acquiring information regarding DPS but place emphasis on information that is personalised to their culture and language. Therefore, more efforts to increase information transfer regarding DPS among women with GDM should be the plan to improve the management of mothers at risk for diabetes. Online platforms such as the use of videos, is a potential source of DPS to explore but to accommodate technical usability, women require culturally relatable content and practical support. In short, findings from this study have significant implications for the improvement of DPS and potential development of DPIs for women living in Malaysia.

## Author contribution

All authors were involved in the discussion of, and formulation of the research questions addressed. IZI and HAZ performed the one-to-one interviews and data collection. Analysis plans and results were discussed and decided by IZI, HAZ, AN, MB and IPN. IZI and HAZ did the initial coding, with themes generated iteratively in team meetings with AN, MB and IPN. IZI prepared the original draft manuscript and IPN edited the final version with feedback from AF and MB. All authors read and approved the final manuscript.

## CRediT authorship contribution statement

**Irmi Zarina Ismail:** Writing – original draft, Visualization, Project administration, Methodology, Formal analysis, Data curation, Conceptualization. **Madeleine Benton:** Writing – review & editing, Supervision, Formal analysis, Conceptualization. **Hafizah Mahamad Sobri:** Resources, Project administration, Methodology, Formal analysis, Data curation. **Anisah Baharom:** Project administration, Methodology, Formal analysis, Data curation, Conceptualization. **Nicola Guess:** Validation, Investigation, Conceptualization. **Kimberley Goldsmith:** Validation, Investigation, Conceptualization. **Iklil Iman:** Project administration, Methodology, Investigation. **Siew Mooi Ching:** Visualization, Validation, Supervision. **Barakatun-Nisak Mohd Yusof:** Visualization, Validation, Project administration. **Nurul Iftida Basri:** Project administration, Methodology, Data curation. **Mazatulfazura Sf Binti Salim:** Investigation. **Faezah Hassan:** Visualization, Investigation, Data curation, Conceptualization. **Helen Murphy:** Investigation. **Angus Forbes:** Writing – review & editing, Validation, Supervision. **Khalida Ismail:** Visualization, Validation, Supervision, Investigation, Funding acquisition. **Boon How Chew:** Validation, Supervision, Investigation. **Iliatha Papachristou Nadal:** Writing – review & editing, Visualization, Validation, Supervision, Conceptualization.

## Funding

The author(s) disclosed receipt of the following financial support for the research, authorship, and/or publication of this article: This work is supported by MYPAIR Grant UK-Malaysia: Joint Partnership Call on Non-Communicable Diseases (Malaysia: JPT.S (BPKl) 2000/011/06/05 (27); UK: MR/T018240/1).

## Declaration of competing Interest

The author(s) declared no potential conflicts of interest with respect to the research, authorship, and/or publication of this article.

## Appendix A Supplementary data

Supplementary material 1 Image 1

Consolidated Criteria for Reporting Qualitative Research (COREQ) checklist Image 2

Consent form Image 3
