# Supplementary material for: Barriers and facilitators to diabetes prevention support for women in Malaysia with gestational diabetes mellitus: A qualitative study
Source: PEC Innov. 2025 Oct 6;7:100438. doi: 10.1016/j.pecinn.2025.100438 (PMC12550234; doi:10.1016/j.pecinn.2025.100438)
Supplement: Supplementary file 1 — Supplementary material 1 [file mmc1.docx]

**Submission checklist**

Before completing the submission of your manuscript, we advise you to read our submission checklist:

- One author has been designated as the corresponding author and their full contact details (email address, full postal address and phone numbers) have been provided.
- All files have been uploaded, including keywords, figure captions and tables (including a title, description and footnotes) included.
- Spelling and grammar checks have been carried out.
- All references in the article text are cited in the reference list and vice versa.
- Permission has been obtained for the use of any copyrighted material from other sources, including the Web.
- For gold open access articles, all authors understand that they are responsible for payment of the article publishing charge (APC) if the manuscript is accepted. Payment of the APC may be covered by the corresponding author's institution, or the research funder.
