# Supplementary material for: Barriers and facilitators to diabetes prevention support for women in Malaysia with gestational diabetes mellitus: A qualitative study
Source: PEC Innov. 2025 Oct 6;7:100438. doi: 10.1016/j.pecinn.2025.100438 (PMC12550234; doi:10.1016/j.pecinn.2025.100438)
Supplement: Supplementary file 3 — Consent form [file mmc3.docx]

INFORMED CONSENT FORM

Title of Study: Barriers and facilitators to diabetes prevention support for women in Malaysia with gestational diabetes mellitus: A qualitative study)

By signing below, I confirm the following:

- I have been given oral and written information for the above study and have read and understood the information given.
- I have had sufficient time to consider participation in the study and have had the opportunity to ask questions and all my questions have been answered satisfactorily.
- I understand that my participation is voluntary, and I can at any time free withdraw from the study without giving a reason and this will in no way affect my future treatment. I am not taking part in any other research study at this time. I understand the risks and benefits, and I freely give my informed consent to participate under the conditions stated. I understand that I must follow the study doctor’s (investigator’s) instructions related to my participation in the study.
- I understand that study staff and their international collaborators, qualified monitors and auditors, the sponsor or its affiliates, and governmental or regulatory authorities, have direct access to my medical record in order to make sure that the study is conducted correctly and the data are recorded correctly. All personal details will be treated as STRICTLY CONFIDENTIAL.
- I will receive a copy of this subject information/informed consent form signed and dated to bring home.
- I agree/disagree* for my family doctor to be informed of my participation in this study. *(*delete which is not applicable)*
- I would like/not like to be informed of the study findings. *(*delete which is not applicable)*
- I also agree for the discussion during the interview to recorded (for data collection and data analysis purpose) in the form of *(*Please tick where applicable)*

Audio recording only

Both audio and video recording

**Subject:**

| Signature: ___________________________ | I/C number: ___________________________ |
| --- | --- |
| Name: ______________________________ | Date: _________________________________ |

**Investigator conducting informed consent:**

| Signature: ___________________________ | I/C number: ___________________________ |
| --- | --- |
| Name: ______________________________ | Date: _________________________________ |

**Impartial witness:**

| Signature: ___________________________ | I/C number: ___________________________ |
| --- | --- |
| Name: ______________________________ | Date: _________________________________ |
